# Effects of Nutrition and Physical Activity Lifestyle Interventions on Childhood Obesity

**DOI:** 10.3390/nu17132100

**Published:** 2025-06-25

**Authors:** Ahmad Alkhatib

**Affiliations:** College of Life Sciences, Birmingham City University, City South Campus, Edgbaston, Birmingham B15 3TN, UK; drahmadalkhatib@gmail.com or ahmad.alkhatib@bcu.ac.uk

Obesity is a disease and a risk factor of multiple debilitating chronic conditions including type-2 diabetes (T2D), hypertension and cardiovascular disease. The rates of adult, adolescent and childhood obesity are all projected to at least double by 2050 [1,2]. Several obesity-related diseases are difficult to treat using glucose-lowering medications, which necessitate appropriate lifestyle interventions [3,4]. Obesity-related cardiometabolic diseases in children and adolescents are now becoming more prevalent, which necessitates targeted interventions for both obesity and its comorbidities.

This Special Issue focused on obesity preventative interventions, through changing lifestyle nutritional eating behaviours, physical activity models and other psychosocial modifiable factors that are tailored to various age groups of children and young people. It also integrated the understanding of lifestyle determinants in children and family context, whether through epidemiological analyses, conceptual models, innovative assessments, nutritional supplements or age-specific interventions and guidelines that inform policy and practice. Nine excellent articles were published that covered these diverse topics while reaching a remarkable 20k views and 17 citations in less than a year since being published.

Three excellent articles from Germany, Spain and Poland provided a European perspective on the understanding of lifestyle behaviours and associated determinants through analyses of large data sets in children and adolescents in schools, communities and home settings [Contribution 1–3]. Overweight and obesity in 1956 German primary school children (7 years old) were analysed using binary logistic regression for their determinants using onsite anthropometrics and parental questionnaires on lifestyle, health, and socioeconomic parameters [Contribution 1]. Results showed that the father and mother’s illness, overweight and obesity status, physical inactivity, smoking (including during pregnancy), being unemployed or having a low income, were all explanatory factors in their children’s overweight and obesity status, while children’s history of being breast-fed by their mother, screen time and physical inactivity were all main determinants of children overweight and obesity status. Interestingly, screen time was also highlighted as an obesity-related factor in 653 Spanish pre-schoolers (6 years old), whose BMI was associated with screen time with physical inactivity, and poor adherence to a healthy Mediterranean diet [Contribution 2]. A cross-sectional analysis of nutritional behaviour of 506 Polish adolescents, following a 9-month extra-curricular physical activity programme “athletics for all”, found better nutritional knowledge (e.g., calorie count, nutrient items), and improved physical fitness in both males and females [Contribution 3]. The study provided insight into how mass population physical activity intervention interlinks with positive lifestyle behaviours to tackle childhood obesity.

Collectively, cross-sectional and intervention studies demonstrated how childhood obesity prevention starts before birth by addressing parents’ lifestyle behaviours including smoking and sedentariness during pregnancy and child infancy years. Monitoring early childhood sedentary behaviour at both home and school is essential since sedentary-related risks and physiological implications continue through the life course [5,6]. In adolescence years, developing physical fitness through an emphasis on sports becomes essential since it reflects well on other lifestyle nutrition and education behaviours. However, parents’ influence on their children’s nutrition behaviour remains a crucial psychosocial factor throughout adolescence years, especially nutrition knowledge, meal quality and frequency adherence. A deep analysis of parents’ feeding practices lasting 16 weeks in 241 African American adolescents, found that parents’ responsiveness and responsibility, measured by “Authoritative Parenting Index”, was associated with increased frequency and quality of family mealtimes [Contribution 4]. Such insights into adolescents’ nutrition behaviours within minority ethnicities emphasises how parents’ engagement is needed when designing effective multicomponent childhood obesity interventions.

Minority ethnicities within high-income countries such as the US and UK have a higher prevalence of both obesity and associated comorbidities including T2D, hypertension, fatty liver and cardiovascular disease. Therefore, the perspectives and experiences of minority ethnicities should also be considered as part of health policies on childhood obesity [7]. In fact, our comprehensive review provided a guideline summary on childhood obesity interventions in minority ethnicities [Contribution 5]. It synthesised extensive evidence to highlight the following key recommendations: (a) objective obesity assessments which consider ethnic-specific BMI cut-off points (b) monitoring obesity secondary cardiometabolic outcomes beyond relying solely on BMI and (c) cultural contextualisation of nutrition, physical activity and other lifestyle behaviours and (d) co-production of effective lifestyle interventions.

Exploring lifestyle behaviours in adolescents in this Special Issue has brought to attention the eating disorder spectrum including anorexia nervosa, which could turn into a pathological obsession with healthy eating behaviour defined as “orthorexia nervosa” (ON) [Contribution 6]. The study reported that individuals suffering from eating disorders, losing weight, exercising heavily, developing relationship problems, and suffering from body dysmorphic disorder are at high risk of developing ON. A significant correlation was found between ON, BMI, and gender, which should be considered when implementing interventions. In this adolescent age group, monetary rewards can be an effective in achieving physical activity and nutrition healthy targets without negatively affecting their knowledge and attitude [Contribution 7]. The latter randomised trial used adherence scores for diet intake, and measuring daily steps, suggesting a “gain-framed incentives” when children are incentivised is superior to “loss-framed” in improving adherence to paediatric obesity weight-loss treatments. The schools, where children spend most of their time provide an excellent setting to integrate intensive multi-component exercise and nutrition behavioural changes into the curriculum, including direct obesity-related physiological measurements and survey questionnaires. This was perfectly shown in the “THINK” trial, a nutrition and exercise middle-school curriculum intervention conducted in Miami, US [Contribution 8]. The program was a good example of how primary and secondary obesity and comorbidities can be ameliorated in a multi-ethnic population, while integrating sustainable healthy behavioural changes.

Another clinical trial highlighted how nutraceutical supplementation combining prebiotics and antioxidants improves T2D metabolic profile (glucose, lipids, kidney function) following a 60-day supplementation in Thai patients with T2D [Contribution 9]. The cultural acceptability of the supplement was key to achieving excellent adherence, especially its locally derived ingredients (e.g., Riceberry rice, inulin rice fibre). Further research is required to explore how supplements alongside other dietary foods, physical activity and contextualised behavioural components affect childhood obesity and associated diseases.
